# A Novel Active Polyphase Filter Employing Frequency-Dependent Image Rejection Enhancement Technique

**DOI:** 10.3390/mi16010065

**Published:** 2025-01-07

**Authors:** Yue Yin, Haobo Qi, Haodong Lu, Ziting Feng, Jiayu He, Xinbing Zhang, Lei Li, Xiaofei Qi, Xiyuan Feng

**Affiliations:** 1 School of Microelectronics, Northwestern Polytechnical University, No. 1 Dongxiang Road, Chang’an District, Xi’an 710129, China; yinyue@nwpu.edu.cn (Y.Y.); qihaobo@mail.nwpu.edu.cn (H.Q.); haodonglu@mail.nwpu.edu.cn (H.L.); ztfeng@mail.nwpu.edu.cn (Z.F.); hejiayv@mail.nwpu.edu.cn (J.H.); xinbing_zhang@mail.nwpu.edu.cn (X.Z.); li13313219079@outlook.com (L.L.); 2 School of Information Science and Technology, Northwest University, No. 1 Xuefu Road, Chang’an District, Xi’an 710127, China; qixf@nwu.edu.cn

**Keywords:** polyphase filter, complex filter, image rejection, low intermediate frequency receiver, notch filter, enhancement technique

## Abstract

In low intermediate frequency (low-IF) receivers, image interference rejection is one of the core tasks to be accomplished. Conventional active polyphase filters (APPFs) are unable to have a sufficient image rejection ratio (IRR) at high operating frequencies due to the degradation of the IRR by the amplitude and phase imbalances produced by the secondary pole. The proposed solution to the above problem is a frequency-dependent image rejection enhancement technique based on secondary pole compensation. By adjusting the dominant pole frequency of the high-pass filter (HPF) appropriately, the proposed technique can theoretically completely reject the image interference signal even in the presence of the secondary pole. The proposed APPF is simulated and fabricated in a 180-nm CMOS process. The simulation results show that the proposed technique can improve the IRR of the APPF by more than 30 dB at the operating frequency of hundreds of MHz. The measured IRR is better than −31 dB at the frequency from 95 to 105 MHz. Unlike conventional schemes, the proposed design is from the perspective of frequency correlation, which makes the operating frequency no longer limited by the secondary pole frequency. In addition, the proposed design also has an excellent IRR for quadrature input signals with phase imbalance.

## 1. Introduction

Nowadays, wireless communication systems pursue higher carrier frequency and wider channel bandwidth to achieve high data rates and low transmission delay [1,2,3,4]. Among the current mainstream receiver architectures, the superheterodyne architecture receiver requires multiple down-conversion processing and off-chip high-performance filters. This does not meet the increasingly demanding size, weight, and power (SWaP) requirements for receivers in applications [5,6]. In addition, superheterodyne architecture receivers are not suitable for broadband applications due to their inherent narrow-band characteristics. Direct down-conversion architecture receivers, consisting of zero intermediate frequency (zero-IF) and low intermediate frequency (low-IF) architecture receivers, are more in line with the requirements of existing and future wireless communication applications for receiver chips [7,8,9,10]. The zero-IF receivers convert the incoming radio frequency (RF) signals directly to the baseband signals, while the low-IF receivers convert the RF signals to intermediate frequency (IF) signals. Zero-IF receivers face problems such as in-phase quadrature (I/Q) imbalance, local oscillator (LO) leakage, direct current (DC) offset, and flicker noise [11]. Furthermore, the zero-IF receiver has one more IF signal processing path that contains a broadband low-pass filter (LPF), a high-accuracy programmable gain amplifier (PGA), and a costly off-chip high-performance analog-to-digital converter (ADC) than the low-IF receiver. To sum up, compared to other architectures, the low-IF architecture receiver has excellent overall performance in terms of received signal quality, signal bandwidth, chip area, and power consumption [12].

The low-IF receiver must have a strong ability to reject image frequency signals. Otherwise, after down-conversion, image frequency noise and potential interfering signals will overlap with the desired signal, severely degrading the signal-to-noise ratio (SNR) of the receiver [13]. There are currently two schemes for image rejection in low-IF receivers. On the one hand, image rejection can be achieved by adding a band-pass filter (BPF) that places the desired frequency in the passband and the image frequency in the stopband before down-conversion. Due to the relatively low-quality factor of the on-chip BPF, the IF must be high enough to achieve a great image rejection ratio (IRR). For example, 7 GHz as in [14,15] or up to 16 GHz as in [16]. However, such a high operating frequency poses significant challenges to the receiver IF module, including the channel selection filter, PGA, and ADC. Overall, the use of a BPF before down-conversion is not a cost-effective and high-performance solution for image rejection in low-IF receivers.

On the other hand, image rejection can be achieved by using a complex filter after down-conversion. In the complex frequency domain, the complex filters can be configured as the complex band-pass filters (CBPFs) or the complex band-stop filters (CBSFs). Few CBPFs operate at frequencies above 100 MHz [17]. This cannot cope with the increasing IF frequencies of low-IF receivers. For example, several low-IF receivers have IF signals with frequencies up to hundreds of MHz or even several GHz [18]. On the contrary, several CBSFs have been proposed to meet the IRR requirements of low-IF receivers at relatively high IF frequencies [19,20]. Almost all existing schemes are passive topologies based on multistage RC polyphase filters (RC-PPF) or transformer-based couplers. The passive topologies are relatively simple to achieve image rejection at high frequencies, but high insertion loss and a large silicon area are unavoidable problems [21,22]. In addition, to address the possible IRR degradation due to process, voltage, and temperature (PVT) variations in the RC-PPF [23], conventional solutions for on-chip passive component tuning include capacitor or resistor arrays, which allow only discrete tuning and limited minimum resolution of tuning. Although active polyphase filters (APPFs) are also negatively affected by PVT variations, APPFs have the general advantages of high signal gain, compact size, and easy tuning compared to passive schemes [24]. However, unavoidable non-ideal factors such as device parasitic parameters and layout asymmetry can lead to severe gain and phase imbalances, which degrade the IRR at high frequencies. To improve the operating frequency of the APPF, the negative effect of the secondary pole on the rejected frequency is mitigated by optimizing the parasitic capacitance of the key node. In addition, the overall layout of the APPF is made more symmetrical to reduce quadrature imbalance. Unfortunately, since parasitic capacitance cannot be eliminated, the conventional APPF optimization scheme becomes less effective or even completely ineffective as the operating frequency further increases.

In this paper, a novel APPF is proposed employing the frequency-dependent image rejection enhancement technique based on secondary pole compensation in the 180 nm CMOS process. Unlike conventional schemes, the operating frequency of the proposed APPF is no longer limited to the secondary pole frequency. Through the analysis of the theoretical model, it is proved that the proposed enhancement technique can fundamentally remove the negative effect of the secondary pole on image rejection. Meanwhile, because the proposed APPF is tunable, great IRRs still can be achieved when the quadrature input signals have phase imbalances, as well as when there are PVT variations. The simulation results show that the proposed image rejection enhancement technique improves the IRR by more than 30 dB in the single-stage circuit at the operating frequency of 100–300 MHz. In addition, the proposed APPF can be cascaded in multiple stages to achieve a better IRR and wider bandwidth, and each stage can be tuned independently to achieve optimal overall performance. The simulation result of the two-stage APPF shows the above conclusion. The measured IRR of −31 dB over the 95–105 MHz frequency band confirms that the proposed enhancement technique is suitable for improving the performance of APPFs at high operating frequencies.

The remaining sections of this paper are organized as follows: Section 2 presents the theoretical model of the APPF and the proposed image rejection enhancement technique; the circuit implementations of the proposed APPF are presented in Section 3; the simulation and measurement results are presented in Section 4; Section 5 compares the simulation and measurement results and analyzes the possible reasons for the discrepancies; and finally, the conclusions are drawn in Section 6.

## 2. Circuit Analysis

### 2.1. Theoretical Model of APPF

**Figure 1:**
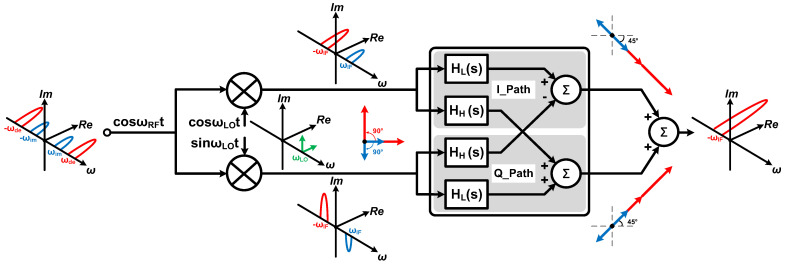
Theoretical model of APPF.

Figure 1 shows the theoretical model of the APPF. After the quadrature down-conversion, the desired signal and the image interference are symmetric about the origin in the complex frequency domain and have a completely opposite phase order. In essence, the APPF is a complex notch filter. According to its theoretical model, the transfer function *H(s)* can be written as

(1) $$\begin{array}{cc} H\left(s\right) & =\frac{I_{O}(s)+jQ_{O}(s)}{I_{I}(s)+jQ_{I}(s)}=\frac{\left[I_{I}(s)H_{L}(s)-Q_{I}(s)H_{H}(s)\right]+j\left[I_{I}(s)H_{H}(s)+Q_{I}(s)H_{L}(s)\right]}{I_{I}(s)+jQ_{I}(s)}\\ & =H_{L}(s)+jH_{H}(s) \end{array}$$

where *H_L_(s)* and *H_H_(s)* are the transfer functions of the LPF and high-pass filter (HPF), respectively. The mixed signals consisting of the desired signal and the image interference pass through the LPFs and HPFs in the I/Q channels. The transfer function of the APPF consisting of first-order LPFs and first-order HPFs can be rewritten as

(2) $$H\left(s\right)=A_{L}\frac{\omega_{L}}{s+\omega_{L}}+jA_{H}\frac{s}{s+\omega_{H}}$$

where *A_L_*, *A_H_*, *ω_L_*, and *ω_H_* are the gains and the pole frequencies of the LPF and HPF, respectively. At the pole frequencies of the LPF and HPF, the phase of the signal lags and leads by 45°, respectively. After combining the output signals of the LPFs and HPFs in the I/Q channels as shown in Figure 1, the image interference in the mixed signal can theoretically be completely rejected, and the desired signal is amplified √2 times. The corresponding mathematical proof can be written as follows:

(3) $$H\left(s=j\omega_{IF}\right)=A\frac{\omega_{IF}}{j\omega_{IF}+\omega_{IF}}+jA\frac{j\omega_{IF}}{j\omega_{IF}+\omega_{IF}}=A\frac{\omega_{IF}-\omega_{IF}}{j\omega_{IF}+\omega_{IF}}=0$$

(4) $$H\left(s=-j\omega_{IF}\right)=A\frac{\omega_{IF}}{-j\omega_{IF}+\omega_{IF}}+jA\frac{-j\omega_{IF}}{-j\omega_{IF}+\omega_{IF}}=A\frac{\omega_{IF}+\omega_{IF}}{-j\omega_{IF}+\omega_{IF}}=A\frac{2}{1-j}$$

*ω_IF_* represents the frequency of the image interference in the complex frequency domain, and −*ω_IF_* represents the frequency of the desired signal. In addition, the gains and pole frequencies of the LPF and HPF must satisfy the operating conditions of *A* = *A_L_* = *A_H_* and *ω_IF_* = *ω_L_* = *ω_H_*. Under such ideal operating conditions, the IRR, defined as the ratio of the signal strength of the image interference to the signal strength of the desired signal, is 0, as shown in Equation (5).

(5) $$IRR(\omega_{IF})=\frac{\left|H\left(s=j\omega_{IF}\right)\right|}{\left|H\left(s=-j\omega_{IF}\right)\right|}=0$$

**Figure 2:**
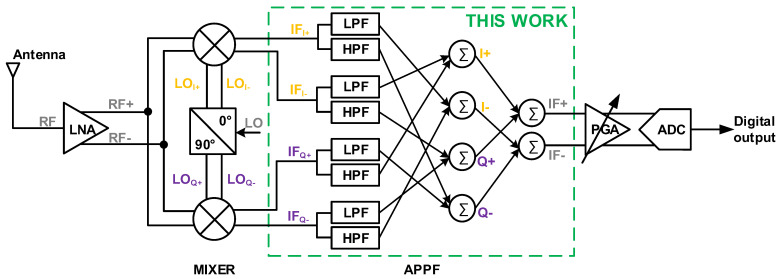
Block diagram of a typical low-IF receiver.

**Figure 3:**
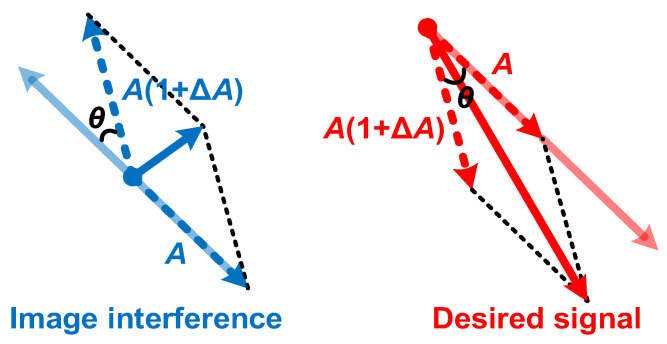
Effect of amplitude and phase imbalance on image interference and desired signal.

Figure 2 shows the block diagram of a typical single-quadrature low-IF receiver that uses an APPF in the IF processing module to reject image interference and slightly amplify the desired signal. Ideally, the image interference can be rejected to null. However, in the actual circuit, there are non-ideal factors such as the parasitic capacitance in the APPF and the less-than-perfect input signal to the APPF, which lead to the degradation of the image rejection effect. Figure 3 illustrates the principle of image rejection degradation due to the amplitude imbalance ∆*A* and phase imbalance *θ* introduced by non-ideal factors. Taking into account the amplitude and phase imbalances, the IRR can be expressed as

(6) IRR=Signalimage→Signaldesired→=A2+A1+ΔA2+2A21+ΔAcosπ−θA2+A1+ΔA2+2A21+ΔAcosθ=1+ΔA2−21+ΔAcosθ+11+ΔA2+21+ΔAcosθ+112

**Figure 4:**
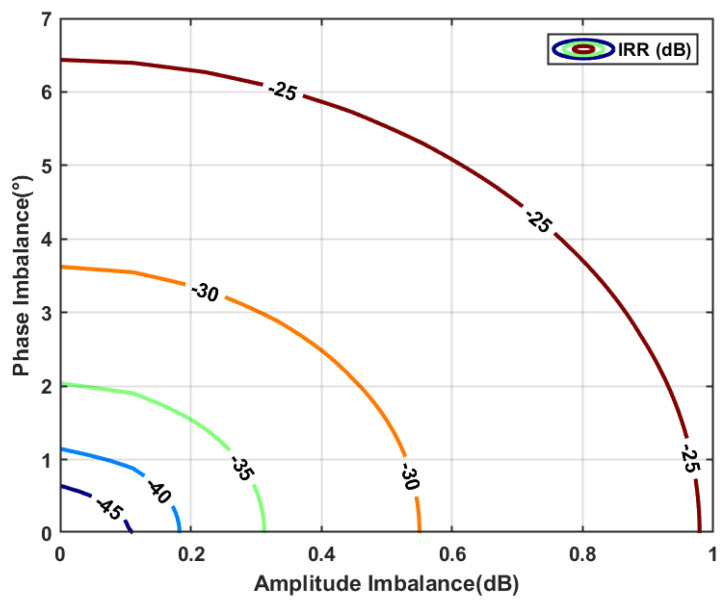
IRR under various amplitude and phase imbalances.

**Figure 5:**
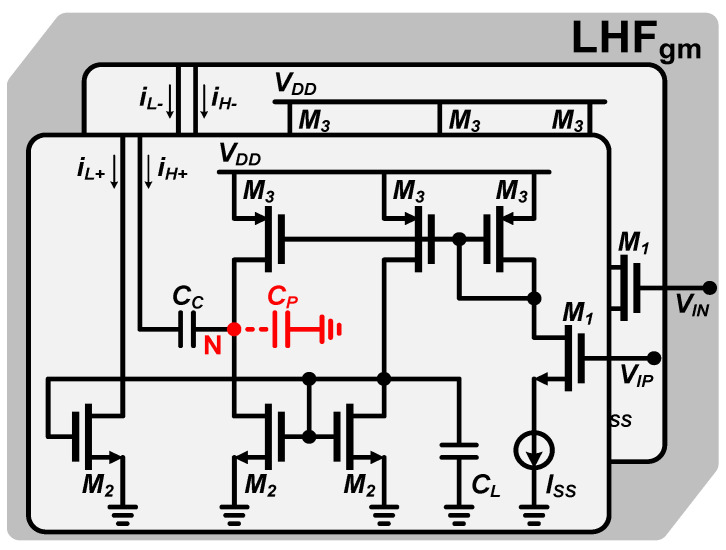
LHF topology in a conventional APPF [24].

**Figure 6:**
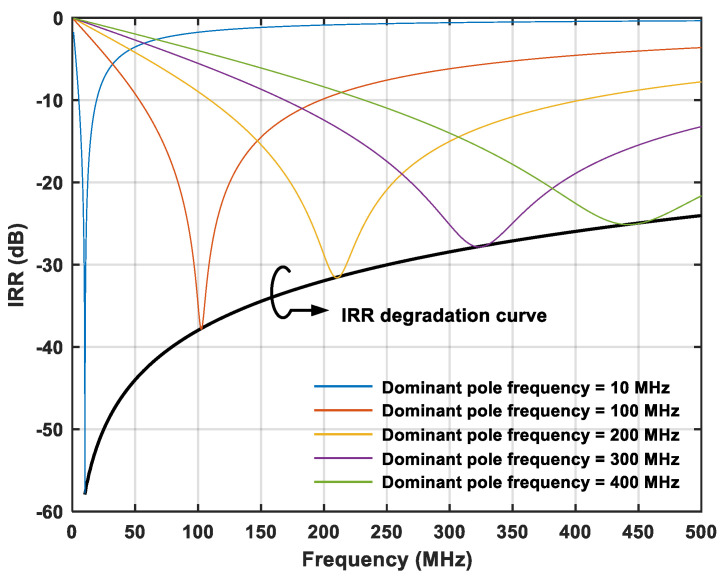
Degradation trend of IRR along with increasing operating frequency.

Figure 4 shows the results of the IRR under various amplitude and phase imbalances, and the numbers on the curves denote the IRR values in decibels. A slight imbalance in amplitude or phase will cause a serious degradation of the IRR. Figure 5 shows the topology of the low-and-high-pass filter (LHF) unit, which consists of low-pass and high-pass transconductance modules in a conventional APPF [24]. Compared to the ideal LPF and HPF transfer functions, the LHF transfer function implemented in the actual circuit deviates from the ideal mathematical model due to the parasitic effect of the physical components. The secondary pole generated by the parasitic capacitance *C_P_* at node N is the most serious inherent non-ideal factor in the APPF. The mismatches of gains and pole frequencies between the LPFs and HPFs caused by *C_P_* will directly result in amplitude and phase imbalances. After considering the secondary pole, the transfer function *H(s)* of the APPF in Equation (2) can be rewritten as

(7) $$\begin{array}{cc} H\left(s\right) & =A_{L}\frac{\omega_{L}}{s+\omega_{L}}+j\left(A_{H}\frac{\omega_{P}}{s+\omega_{P}}-A_{H}\frac{\omega_{H}}{s+\omega_{H}}\right)\\ & =A_{L}\frac{\omega_{L}}{s+\omega_{L}}+j\left(A_{H}\frac{C_{L}-C_{P}}{C_{L}}\right)\left(\frac{s}{s+\omega_{H}}\cdot \frac{\omega_{P}}{s+\omega_{P}}\right) \end{array}$$

where *ω_P_* and *C_L_* are the secondary pole frequency and the main capacitance, respectively. In Figure 5, the formula for the dominant pole frequency and the secondary pole frequency are *ω_L_* = *ω_H_* = *g_m2_*/*C_L_* and *ω_P_* = *g_m2_*/*C_P_*, respectively, where *g_m2_* is the transconductance value of transistor M_2_. The gain of the HPF is no longer *A_H_*, but becomes the product of *A_H_* and the coefficient (*C_L_* − *C_P_*)/*C_L_*. Since the coefficient (*C_L_* − *C_P_*)/*C_L_* is constantly less than one, the gain of the LPF *A_L_* is no longer equal to the gain of the HPF *A_H_*(*C_L_* − *C_P_*)/*C_L_*. It can be seen from (7) that *C_P_* and *ω_P_* cause the gain and pole frequency mismatches of the APPF, which will introduce amplitude and phase imbalances. *C_P_* at node N in Figure 5 is composed of the gate-drain parasitic capacitance *C_gd_*, the gate-source parasitic capacitance *C_gs_*, the drain junction capacitance *C_jd_*, and the source junction capacitance *C_js_* of the MOSFETs. Furthermore, the layout and routing will also introduce some of the parasitic capacitance at node N. Taking the 180 nm CMOS process as an example, on the premise that the overall power consumption is acceptable, *g_m2_* is set at 500 μS. After using the relatively short transistors to reduce the parasitic capacitance, the value of *C_P_* is still in the tens of femto-farads (fFs). Reducing the channel length of the transistors further not only does not effectively reduce the value of *C_P_* but also the output resistance of the transistors will be smaller, which can produce parasitic zeros that have a negative effect on image rejection. By setting *C_P_* to 20 fF, *ω_P_* can be obtained in the vicinity of 4 GHz, which is close to the limit of the 180 nm CMOS process. Figure 6 shows the trend of IRR degradation with increasing operating frequency. At relatively high operating frequencies, the ratio of *C_P_* to *C_L_* increases, and the frequencies of *ω_L_* and *ω_H_* are closer to the frequency of *ω_P_*. These lead to increased gain and pole frequency mismatches, which negatively affect and severely degrade the image rejection ability of the APPF.

### 2.2. Frequency-Dependent Image Rejection Enhancement Technique Based on Secondary Pole Compensation

In view of the unavoidable negative influence of *ω_P_* in the APPF, a novel frequency-dependent image rejection enhancement technique based on the secondary pole compensation is proposed from the perspective of frequency correlation, instead of pursuing the ultimate optimization of parasitic parameters. After considering the gain and pole frequency mismatches caused by *ω_P_* and *C_P_*, the IRR of the APPF can be rewritten as

(8) IRR(ωIF)=H(s=jωIF)H(s=−jωIF)=ALωL−1ωP−ωHωIF+2ωPωP−ωHωH−AHALωIF+jωIFωP+ωHωP−ωHωL−AHALωIFjωIF+ωL−1ωP−ωHωIF+2ωP+ωHωP−ωHjωIF+ωPωHωP−ωHALωL−1ωP−ωHωIF+2ωPωP−ωHωH+AHALωIF−jωIFωP+ωHωP−ωHωL+AHALωIF−jωIF+ωL−1ωP−ωHωIF−2ωP+ωHωP−ωHjωIF+ωPωHωP−ωH

|*H(s=jω_IF_)*|, i.e., the signal strength at the image interfering frequency must be minimal to achieve an optimum IRR. Under the condition of *A* = *A_L_* = *A_H_*, the image interference strength can be obtained from the following equation:

(9) Hs=jωIF=AωL−1ωP−ωHωIF+2ωPωP−ωHωH−ωIF+jωIFωP+ωHωP−ωHωL−ωIFjωIF+ωL−1ωP−ωHωIF+2ωP+ωHωP−ωHjωIF+ωPωHωP−ωH

It can be seen from (9) that *ω_IF_*,* ω_L_*,* ω_H_*, and *ω_P_* should satisfy Equation (10).

(10) −1ωP−ωHωIF+2ωPωP−ωHωH−ωIF=0ωP+ωHωP−ωHωL−ωIF=0

Since the other set of solutions has no practical significance, (11) is the only realizable solution to Equation (10).

(11) $$\omega_{IF}=\omega_{H}=\frac{\omega_{P}+\omega_{IF}}{\omega_{P}-\omega_{IF}}\omega_{L}$$

It is obvious that *ω_IF_ = ω_H_ = ω_L_* in the previous APPF design cannot satisfy the requirements of Equation (11). In a conventional APPF, efforts are made to increase *ω_P_* to infinity by optimizing the parasitic effect. However, the manufacturing process always has parasitic parameters that result in a constant *ω_P_*, so the degradation of IRR in a conventional APPF is aggravated with the increase in *ω_IF_*. On the contrary, the proposed frequency-dependent image rejection enhancement technique based on the second pole compensation satisfies the proportional relationship in Equation (11) by tuning *ω_H_* and completes the cancellation compensation of the negative effects caused by *ω_P_*.

**Figure 7:**
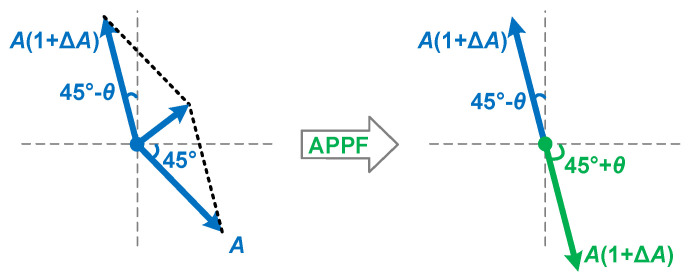
Compensation mechanism of the proposed enhancement technique.

**Figure 8:**
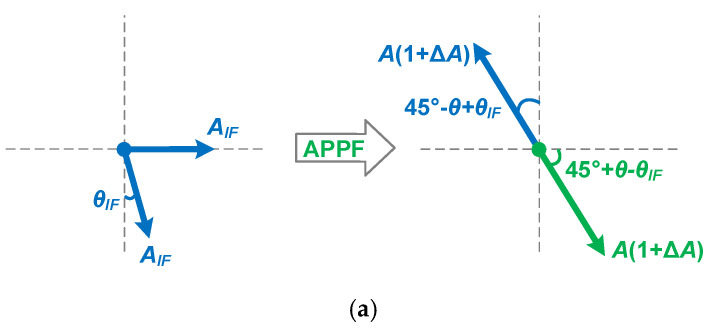

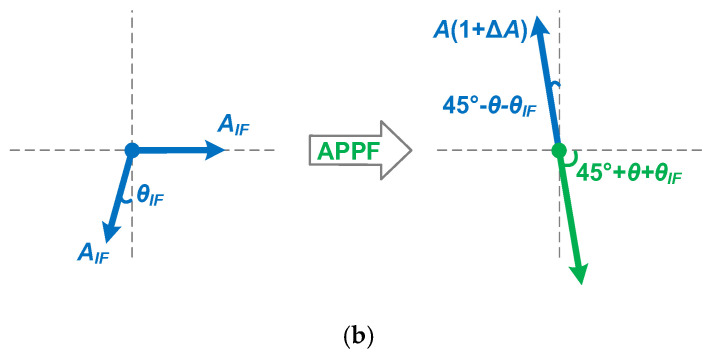
Processing mechanism to phase imbalance of the input signal: (**a**) the quadrature input signal is less than 90°; and (**b**) the quadrature input signal is greater than 90°.

Figure 7 demonstrates the compensation mechanism for the negative effects of the secondary pole. The traditional *ω_IF_ = ω_H_ = ω_L_* condition causes the image interference phase to be lagged by 45° at the dominant pole frequency *ω_L_* of the LPF. However, in the HPF, due to the amplitude imbalance ∆*A* and phase imbalance *θ* introduced by the secondary pole, the phase shift at the operating frequency *ω_IF_*, that is, the dominant pole frequency *ω_H_* of the HPF, is no longer 45°. And, the amplitude characteristic at *ω_IF_* is also no longer consistent with that of the LPF. As a result, the image interference cannot be effectively rejected. In the proposed enhancement technique, the operating frequency *ω_IF_* is set to the new dominant pole frequency *ω_H_* of the HPF, and the dominant pole frequency *ω_L_* of the LPF remains unchanged. In this case, the amplitude characteristic and the phase shift at *ω_IF_* of the LPF are *A*(1 + ∆*A*) and 45° − *θ*, respectively. The combination of the output signals processed by the LPF and the HPF can completely eliminate the image interference. In addition, IF signals at the input of the APPF may have amplitude and phase imbalances. Phase imbalance is more difficult to deal with than amplitude imbalance because the latter can be eliminated by adding circuits such as limiters, buffers, etc., before the APPF. Figure 8 shows the processing mechanism of the proposed enhancement technique in response to the phase imbalance of the input signal. There are two possible cases where the phase difference between the I channel and Q channel signals at the input of the APPF is less than or greater than 90°. Compared with traditional schemes, the proposed enhancement technique can better overcome the degradation of the image rejection ability of the APPF when the phase difference of the input quadrature signal is not equal to 90°. Because the proposed technique can simultaneously handle the phase imbalance *θ_IF_* and the phase imbalance *θ* and can relatively well handle the amplitude imbalance ∆*A*. Based on the above theoretical analysis, the proposed technique can effectively improve the image rejection ability of APPFs, especially at high operating frequencies.

## 3. Circuit Implementation

In APPFs, both *ω_H_* and *ω_L_* are determined by the transistor transconductance and the main capacitance. In contrast to the capacitance value, which can only be tuned discretely by a switched-capacitor bank, the transconductance value can be easily tuned continuously by changing the current flowing through the transistor. Therefore, it is a more reliable circuit implementation scheme to adjust the transistor transconductance value to achieve the proposed image rejection enhancement technique.

**Figure 9:**
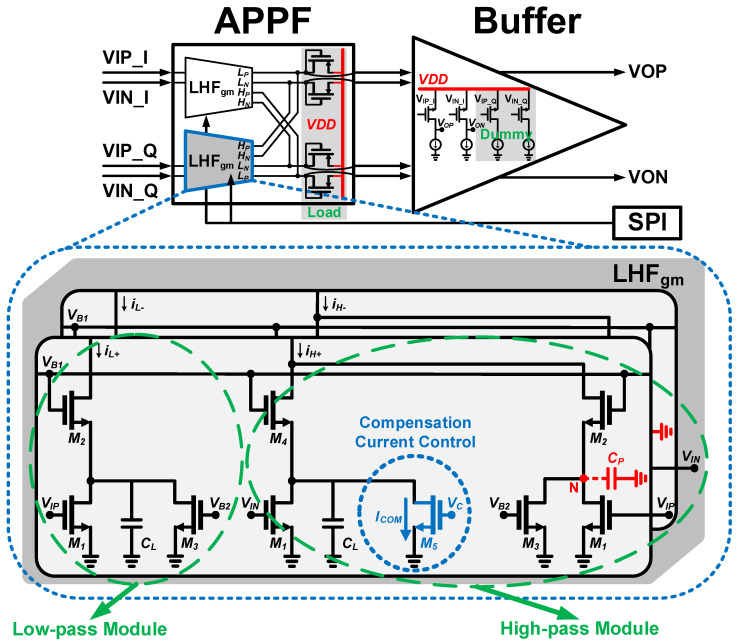
Schematic of the novel single-stage APPF with the proposed enhancement technique.

Figure 9 shows the novel single-stage LHF topology, consisting of low-pass modules and high-pass modules. The low-pass module consists of transistors M_1_, M_2_, M_3,_ and the main capacitor *C_L_*. The output current *i_L_* of the low-pass module is converted to voltage by connecting it to a diode-connected transistor M_Load_. The transfer function of the low-pass module *H_L_(s)* can be written as

(12) $$H_{L}\left(s\right)=\frac{g_{m1}}{g_{mLoad}}\cdot \frac{g_{m2}/C_{L}}{s+g_{m2}/C_{L}}$$

where *g_m1_*, *g_m2_*, and *g_mLoad_* are the transconductance values of M_1_, M_2_, and M_Load_, respectively. The high-pass module consists of transistors M_1_, M_2_, M_3_, M_4_, M_5_, and the main capacitor *C_L_*. After taking into account the parasitic capacitance *C_P_* at the node N, the transfer function of the high-pass module *H_H_(s)* can be derived as

(13) $$\begin{array}{cc} H_{H}(s) & =\frac{g_{m1}}{g_{mLoad}}\cdot \frac{g_{m2}/C_{P}}{s+g_{m2}/C_{P}}-\frac{g_{m1}}{g_{mLoad}}\cdot \frac{g_{m4}/C_{L}}{s+g_{m4}/C_{L}}\\ & =\frac{g_{m1}}{g_{mLoad}}\cdot \frac{s}{s+g_{m4}/C_{L}}\cdot \frac{g_{m2}/C_{P}-g_{m4}/C_{L}}{s+g_{m2}/C_{P}} \end{array}$$

where *g_m4_* is the transconductance value of M_4_. In the novel single-stage LHF topology, *ω_L_*,* ω_H_*, and *ω_P_* are generated by *g_m2_/C_L_*, *g_m4_/C_L_*, and *g_m2_/C_P_*, respectively. In order to implement the proposed enhancement technique, the transistors M_5_ are added to the high-pass modules to introduce the additional compensation currents to change the magnitude of the currents flowing through M_4_. In this way, the required *ω_H_* can be obtained while *ω_L_* is not affected in any way. The transistors M_3_ are added to make the circuit more symmetrical and matched and to help compensate for the phase imbalance *θ_IF_*. In order to satisfy the condition between *ω_H_* and *ω_L_* in (11), *g_m4_* and *g_m2_* in the proposed APPF must satisfy the proportional relationship determined by *ω_P_* and the operating frequency *ω_IF_*, as shown in Equation (14).

(14) $$\frac{g_{m4}}{g_{m2}}=\frac{\omega_{P}+\omega_{IF}}{\omega_{P}-\omega_{IF}}$$

The relationship between the transconductance value *g_m_* of a transistor operating in the saturated region and the current *I* flowing through the transistor is as follows:

(15) $$g_{m}=\sqrt{2\mu_{n}C_{ox}\frac{W}{L}I}$$

where *μ*_n_, C_ox_, and W/L are the mobility of the electrons, the gate oxide capacitance per unit area, and the aspect ratio of the transistor, respectively. It can be seen that by controlling transistor M_5_ to vary the additional compensation current *I_COM_*, the current *I_4_* flowing through transistor M_4_ can be varied to adjust *g_m4_*. Transistors M_2_ and M_4_ have the same physical parameters such as the aspect ratio. The function of *I_COM_* changing with *ω_IF_* can be derived as

(16) $$I_{COM}\left(\omega_{IF}\right)=\left(I_{1}+I_{3}\right){\left(\frac{\omega_{P}+\omega_{IF}}{\omega_{P}-\omega_{IF}}\right)}^{2}-I_{1}$$

where *I_1_* and *I_3_* are the currents flowing through transistors M_1_ and M_3_, respectively. And, *ω_P_* is also an objective constant. Therefore, after determining *ω_IF_*, there is a unique optimal value for *I_COM_* to achieve the ideal image rejection. In addition, the proposed APPF can be cascaded in several stages to achieve a higher IRR and wider bandwidth to meet the requirements of some broadband applications. The different operating frequencies in each stage are obtained by adjusting the main capacitors *C_L_*, and the proposed enhancement technique in each stage can be tuned independently to obtain the optimal overall performance.

## 4. Simulation and Measurement Results

**Figure 10:**
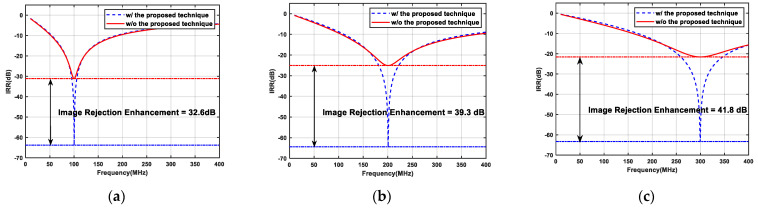
Simulated results of the single-stage APPF with different operating frequencies: (**a**) the operating frequency is 100 MHz; (**b**) the operating frequency is 200 MHz; and (**c**) the operating frequency is 300 MHz.

The 180 nm CMOS process was used to design the proposed novel single-stage APPF. The layout area of the single-stage APPF is 150 × 340 μm^2^. The power consumption simulation result for a single-stage APPF is 1.48 mW with a supply voltage of 1.8 V. The simulated results of the single-stage APPF with different operating frequencies in Figure 10 show that the proposed technique significantly improves the image rejection ability. And, the effect of the image rejection improvement does not degrade with the increase in operating frequency, which effectively solves the shortcoming of the insufficient image rejection ability of the conventional APPF in the high-frequency range. At a frequency of several hundred MHz, the proposed single-stage APPF has an optimum IRR of better than −60 dB.

**Figure 11:**
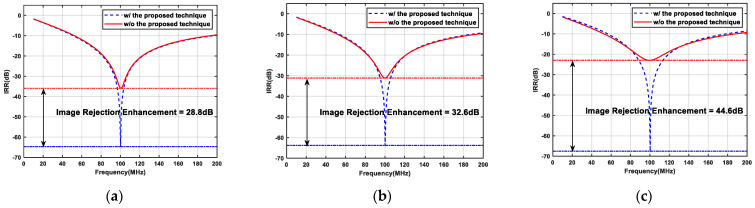
Simulated results of the single-stage APPF with input signals of different phases: (**a**) the quadrature input signal phase is 85°; (**b**) the quadrature input signal phase is 90°; and (**c**) the quadrature input signal phase is 95°.

Figure 11 shows the image rejection effect of APPF when there is a phase imbalance in the quadrature input signal. Without employing the proposed technique, the phase imbalance of the quadrature input signal may be superimposed with the phase imbalance introduced by the secondary pole, which further worsens the IRR. On the contrary, the APPF with the proposed technique has an excellent IRR regardless of whether the phase of the quadrature input signal is greater than or less than 90°, which proves that the proposed enhancement technique can reduce the strict requirement on the input signal phase.

**Figure 12:**
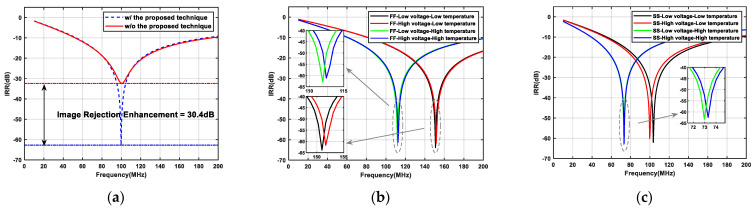
Simulated results of the single-stage APPF at different PVT combinations: (**a**) TT-Typical voltage-Typical temperature; (**b**) four PVT combinations under the FF process corner: FF-Low voltage-Low temperature; FF-High voltage-Low temperature; FF-Low voltage-High temperature; and FF-High voltage-High temperature; and (**c**) four PVT combinations under the SS process corner: SS-Low voltage-Low temperature; SS-High voltage-Low temperature; SS-Low voltage-High temperature; and SS-High voltage-High temperature.

**Figure 13:**
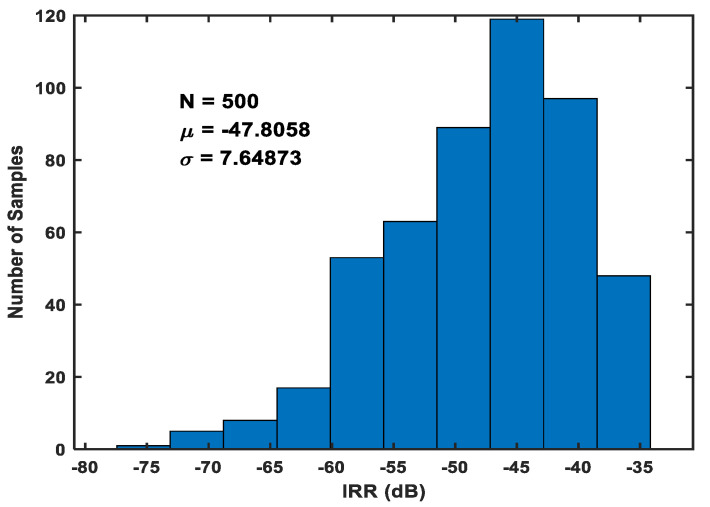
Simulation of the IRR of the single-stage APPF based on 500 Monte Carlo results.

With regard to the PVT variations, nine combinations were selected for simulation. The TT-Typical voltage-Typical temperature combination is set as the control group. The eight experimental groups included FF-Low voltage-Low temperature, FF-High voltage-Low temperature, FF-Low voltage-High temperature, FF-High voltage-High temperature, SS-Low voltage-Low temperature, SS-High voltage-Low temperature, SS-Low voltage-High temperature, and SS-High voltage-High temperature. Figure 12 shows the IRR variations for different PVT combinations, indicating that the IRR can always exceed −60 dB at the operating frequency. Low, typical, and high voltages are set to 1.8 V, 1.62 V, and 1.98 V, respectively. Low, typical, and high temperatures are −40 degrees Celsius, 27 degrees Celsius, and 85 degrees Celsius, respectively. The frequency offset of the operation due to PVT variations can be compensated by adjusting the capacitance or tuning the transconductance by adjusting the tail current. The Monte Carlo simulation results for the IRR are shown in Figure 13. The total number of runs is 500, and the standard deviation of the IRR is observed to be less than 7.65 dB, demonstrating a high robustness.

**Figure 14:**
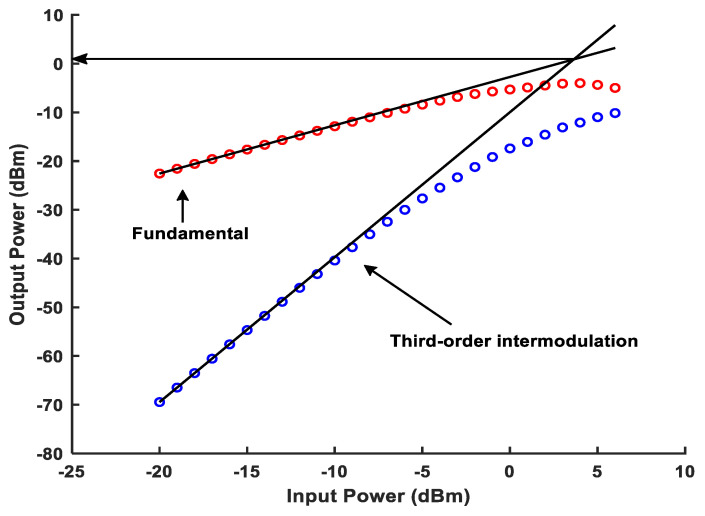
OIP3 simulation result of the proposed single-stage APPF.

**Figure 15:**
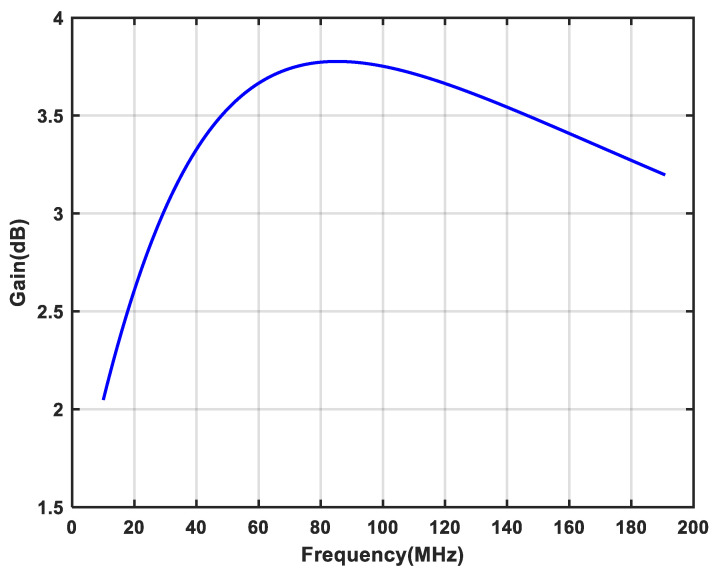
Gain simulation result of the proposed single-stage APPF.

As shown in Figure 14, the simulation result of the output third intercept point (OIP3) of the single-stage APPF is greater than 0.94 dBm. Figure 15 shows the gain simulation result of the single-stage APPF near 100 MHz operating frequency is around 3.75 dB. Furthermore, the input-referred noise (IRN) simulation result of the single-stage APPF is 34.5 μV.

**Figure 16:**
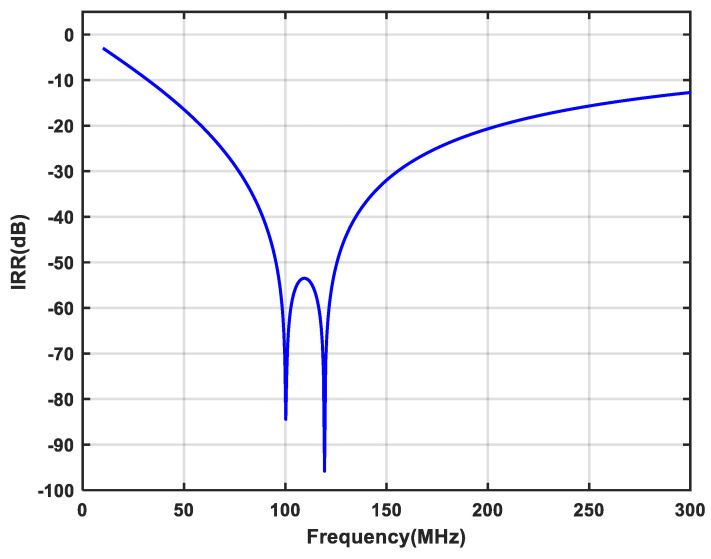
Simulated result of the two-stage APPF employing the proposed enhancement technique.

The proposed enhancement technique can also be used in multistage APPF, where each stage can be tuned independently to improve the image rejection ability of broadband signals. The different rejection frequencies in each stage are obtained by adjusting the main capacitors. Figure 16 shows the simulated result of the two-stage APPF employing the proposed technique. It can achieve an IRR of better than −53.4 dB at a bandwidth of 26 MHz.

**Figure 17:**
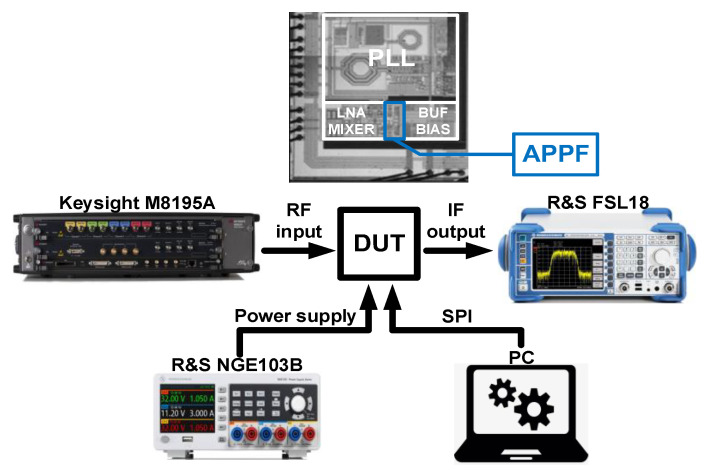
Photograph of the proposed single-stage APPF and the measurement setup.

The 180 nm CMOS process was also used to fabricate the proposed novel single-stage APPF to verify the effectiveness of the frequency-dependent image rejection enhancement technique. The photograph of the fabricated APPF die and the measurement setup is shown in Figure 17. The RF input signal generated by the signal generator is fed into the chip and amplified by a low-noise amplifier (LNA). The phase-locked loop (PLL) generates quadrature LO signals. In the mixer, the amplified RF signal is down-converted into quadrature IF signals by the quadrature LO signals. Then, IF signals flow into the APPF to complete image interference rejection. To drive the measurement instruments, a low-noise on-chip buffer with high linearity is placed after the APPF. The bias circuit provides the appropriate bias voltages for each of the other modules on the chip. Finally, the output signal of the chip is fed into the spectrum analyzer to complete the performance test.

**Figure 18:**
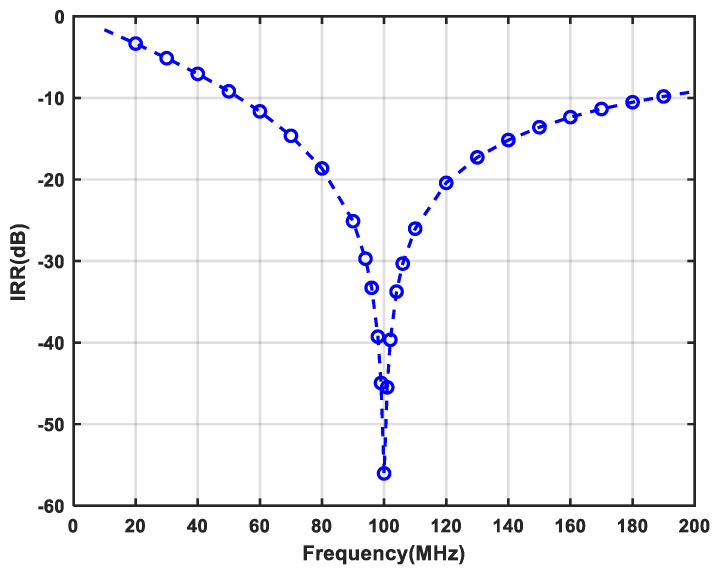
Measured IRR of the proposed single-stage APPF.

**Figure 19:**
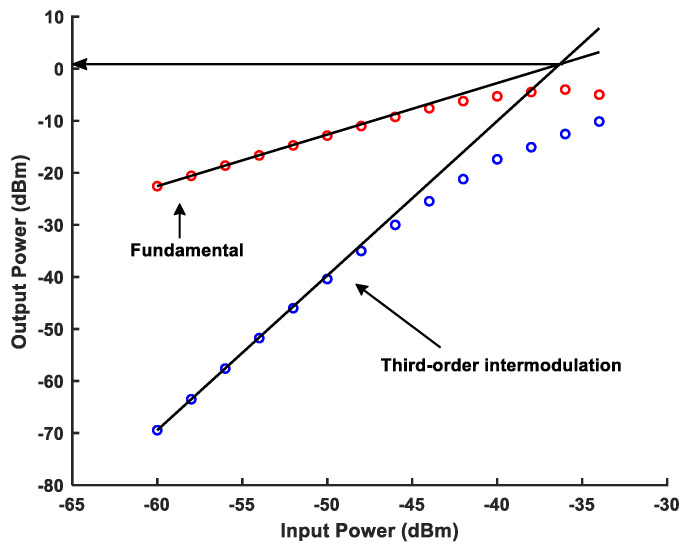
Measured OIP3 at the output of the proposed single-stage APPF.

The core area of the single-stage APPF is 150 × 340 μm^2^. With a supply voltage of 1.8 V, the power consumption is 1.5 mW. Figure 18 shows that the measured IRR of −31 dB can be achieved in the frequency range of 95 to 105 MHz. On the one hand, choosing 10 MHz as the operating bandwidth to measure the IRR is the bandwidth requirement for certain wireless system applications, such as satellite navigation, the Internet of Things (IoT), and mobile communications. On the other hand, an IRR of greater than 30 dB can be obtained from the references [6,17,18] to basically meet the image rejection capability requirements of many wireless communication standards. And, it has an optimum IRR of better than −56 dB. The OIP3 at the output of the APPF is greater than 0.9 dBm, as shown in Figure 19. The gain and the IRN are extrapolated to 3.53 dB and 37.4 μV, respectively.

## 5. Comparative Analysis of Simulation and Measurement Results

Table 1 demonstrates the comparison of the simulated and measured results of each performance of the proposed single-stage APPF. Among them, the simulation and measurement results for the performance of power consumption, area, OIP3, gain, and IRN are in general agreement. Errors in power consumption, area, gain, and IRN are mainly composed of accuracy errors in the simulation model and random errors in the actual test. The OIP3 simulation object only includes the proposed APPF, while the OIP3 test is the output of the whole channel including the LNA, mixer, single-stage APPF, and buffer. The LNA and mixer combination before the APPF has a gain of about 40 dB on the signal, so there is a 40 dB difference in input signal power between the OIP3 simulation and measurement results. The buffer circuit with high linearity after the APPF is a gain approximately equal to 0 dB, so the overall channel circuit linearity is basically determined only by the single-stage APPF. Therefore, the simulation and measurement results of OIP3 are basically the same.

The IRR measurement results show a certain degree of degradation compared to the simulation results. Specifically, the optimal IRR is degraded by 6.7 dB, and the IRR at 10 MHz operating bandwidth is degraded by 0.7 dB. The possible causes of degradation include the following. The first is the control accuracy of the compensation current. In the simulation, the compensation current can be adjusted to the optimal value so as to obtain the optimum IRR, while in the chip, the compensation current is controlled by the serial peripheral interface (SPI), and the resolution of the compensation current value is limited. The second is a slight I/Q channel amplitude and phase imbalances that may exist in the chip, caused by random errors in the I/Q channel devices during production. The third is that the quadrature LO signals generated by the PLL may be less than perfect, with some amplitude and phase imbalances. The proposed design can handle the phase imbalance of the signals input to the APPF well, and the possible amplitude imbalance in the signals will make the IRR slightly degraded. Although the IRR measurement results of the single-stage APPF are negatively impacted by the above factors, it still has an excellent image rejection effect.

In summary, the measurement results are slightly degraded compared to the simulation results, but the differences are within the acceptable normal range. The measurement results strongly support the effectiveness of the proposed technique and prove the application value of the proposed design.

The overall performance of the proposed design is compared with [13,17,18,21,24], as shown in Table 2. In low-IF receivers, image rejection filter schemes realized after down-conversion include the complex filter schemes RC-PPF, Active-RC, CBPF, and APPF. IRR is the core performance parameter of the image rejection filter because the rejection capability is measured by it. Image rejection filters can be cascaded in multiple stages to improve the IRR. Therefore, IRR per stage, a parameter defined as the total IRR divided by the number of stages, is listed to more objectively compare the image rejection capabilities of different designs. In addition, the bandwidth is also an important parameter of the image rejection filter; the selection of the center frequency largely determines the value of the operating bandwidth. The higher the center frequency, the larger the bandwidth. The value of the operating bandwidth is also related to the number of stages. By setting the center frequency of each stage appropriately, the operating bandwidth of the image rejection filter can be effectively expanded. As a key part of the receiver chip, the power consumption, area, OIP3, gain, and IRN of the image rejection filter also have a significant impact on the receiver system.

The proposed APPF has a better IRR and a smaller number of stages compared to other works using more advanced process, which can also be visualized from IRR per stage. The significantly larger operating bandwidths of [13,18] compared to the other works are due to the fact that their center frequencies are chosen at several GHz. The proposed design has an advantage in terms of operating bandwidth with similar center frequencies. Meanwhile, the proposed APPF can work in a higher operating frequency range compared to other active complex filter schemes [17,24]. In terms of power consumption and area, RC-PPFs [18,21] occupy a larger on-chip area and require energy-consuming buffers with good driving capabilities to assist them, although they do not have static power consumption. In the active image rejection filter, the power consumption and area depend mainly on the manufacturing process, supply voltage, and number of stages. Therefore, the proposed work has moderate parameters. With respect to the OIP3 parameters, the proposed design performs generally well and basically satisfies the application requirements of circuits such as the ADC in the wireless system. In addition, the two performance parameters of gain and IRN of the proposed design also perform at relatively excellent levels compared to other works. Compared to the active image rejection filter scheme, RC-PPFs [18,21] have not only no gain but also insertion loss. In summary, the proposed APPF shows better image rejection and excellent overall performance.

## 6. Conclusions

This paper proposes and verifies the frequency-dependent image rejection enhancement technique based on the secondary pole compensation. The proposed technique also has an excellent image rejection effect for quadrature input signals with phase imbalance. The implemented single-stage APPF has an optimum IRR of −56 dB at the operating frequency of 100 MHz. Meanwhile, the proposed APPF also has a gain of 3.53 dB, the desired signal. By cascading multistage APPFs, the bandwidth can be expanded to meet the requirements of broadband applications. In more advanced CMOS process nodes, the APPF operating frequency can be increased up to GHz by utilizing the image rejection enhancement technique proposed in this paper.

## Figures and Tables

**Table 1:** Performance comparison between simulation and measurement results.

Result	Optimum IRR (dB)	IRR (dB) @ Band-Width > 10 MHz	Power (mW)	Area (mm^2^)	OIP3 (dBm)	Gain (dB)	IRN (μV)
Simulation	63.7	31.7	1.48	0.051	0.94	3.75	34.5
Measurement	57	31	1.5	0.051	0.9	3.53	37.4

**Table 2:** Performance summary and comparison.

Parameter	2020 [18]	2020 [21]	2020 [13]	2023 [17]	2005 [24]	This Work	
Process	90 nm CMOS	65 nm CMOS	65 nm CMOS	22 nm FD-SOI	250 nm CMOS	180 nm CMOS	
Type	RC-PPF	RC-PPF	Active-RC	CBPF	APPF	APPF	
Number of stages	3	2	2 ^(2)^	1	4	1	2 ^(2)^
Total IRR (dB)	−30	−40 ^(1)^	−40 ^(2)^	−27	−48	−31	−53.4 ^(2)^
IRR per stage (dB)	−10	−20 ^(1)^	−20 ^(2)^	−27	−12	−31	−26.7 ^(2)^
Bandwidth/Center frequency (MHz)	1850/1580	20/200	2000/3000 ^(2)^	5/60	23.9/18.1	10/100	26/110 ^(2)^
Power (mW)	N/A	N/A	15 ^(2)(3)^	0.099	11	1.5	2.98 ^(2)^
Core area (mm^2^)	0.25	0.19	0.0091 ^(2)^	0.0049	0.95	0.051	0.081 ^(2)^
OIP3 (dBm)	N/A	−6.0 ^(1)^	5.8 ^(2)^	3.4 ^(1)^	14.6 ^(1)^	0.9	−1.55 ^(2)^
Gain (dB)	−14.3	N/A	14 ^(2)(3)^	13.6	6.6	3.53	6.68 ^(2)^
IRN (μV)	N/A	N/A	N/A	107.7	78.1	37.4	58.6 ^(3)^

^(1)^ Deduced parameter. ^(2)^ Simulation result. ^(3)^ With I/Q mixer and buffer.

## Data Availability

The data are contained within the article; further inquiries can be directed to the corresponding author.
